# FALLS AND MOTOR VEHICLE COLLISIONS: A LONGITUDINAL INVESTIGATION OF OLDER DRIVERS

**DOI:** 10.1093/geroni/igz038.1069

**Published:** 2019-11-08

**Authors:** Caitlin N Pope, Pariya Fazeli, Tyler R Bell, Meghana Gaini, Sylvie Mrug, Karlene Ball

**Affiliations:** 1 Center for Injury Research and Policy, The Research Institute at Nationwide Children's Hospital, Columbus, Ohio, United States; 2 University of Alabama at Birmingham, Birmingham, Alabama, United States; 3 Pennsylvania State University, State College, Pennsylvania, United States

## Abstract

Longitudinal research is needed to better understand mobility and aging, as falls and motor vehicle collisions (MVCs) are the top two leading causes of unintentional injury-related deaths for adults 65 and older in the United States. Using a longitudinal sample of older adults, prior falls were assessed as a predictor and moderator of the rate of subsequent MVCs over a 15 year time period. Using a 15-year longitudinal sample of 1,911 older adults recruited from three Maryland State Motor Vehicle Administration (MVA) sites, we conducted group differences and Generalized Estimating Equation (GEE) Poisson regressions. Individuals who reported a fall at baseline were more likely to be female, older, have poorer physical functioning, and reported more situational driving avoidance at baseline compared to those who did not report a fall. Females who reported a fall at baseline had a 2x greater risk rate of subsequently reporting a MVC over the 15 year time period than males. Furthermore, individuals, irrespective of gender, with a prior fall at baseline who drove more days per week over the 15-year time span had a 23% higher risk rate of a subsequent MVC. The current findings further the discussion on aging and mobility as it offers a longitudinal perspective on the association between falls and MVCS. These findings promote the utility of investigating non-traditional driver screening methods to identify drivers who may be at an increased rate for further driving difficulties.

